# Accessing Frank–Kasper Phases via Blending of Architecturally Distinct and Sustainable Sugar-Based Block Co-Oligomers

**DOI:** 10.1021/acs.macromol.5c01479

**Published:** 2025-08-06

**Authors:** Yu-Hung Cheng, Ting-Wei Chang, Taiki Nishimura, Yi-Cheng Lai, Chun-Jen Su, Jane Wang, Takuya Isono, Toshifumi Satoh, Hsin-Lung Chen

**Affiliations:** † Department of Chemical Engineering, 34881 National Tsing Hua University, Hsinchu 300043, Taiwan; ‡ Graduate School of Chemical Sciences and Engineering, 12810 Hokkaido University, Sapporo 060-8628, Japan; § 57815 National Synchrotron Radiation Research Center, Hsinchu 300092, Taiwan; ∥ Faculty of Engineering, 12810 Hokkaido University, Sapporo 060-8628, Japan

## Abstract

Sugar-based block co-oligomers (BCOs), composed of polar oligosaccharide and nonpolar hydrocarbon blocks, have emerged as a promising platform for generating complex, thermally stable nanostructures with ultrasmall feature size, including network and Frank–Kasper (FK) phases. Here, we show that the blending of chemically distinct sugar-based BCOs offers an effective alternative to precision synthesis for tuning self-assembly in this class of sustainable materials. We investigate the binary blends of glucose–(solanesol)_2_ (Glc_1_-(Sol)_2_, G1) and 1,3-(maltose)_2_–(solanesol)_2_ (1,3-(Glc_2_)_2_-(Sol)_2_, CIS), which possess AB_2_ and A_2_B_2_ architectures, respectively. While neat G1 and CIS individually formed dodecagonal quasicrystal (DDQC)/body-centered cubic (BCC) and lamellar/hexagonally perforated layer morphologies, their blends exhibited emergent micelle packing structures, including FK σ and A15 phases. Notably, thermal processing of these blends further induced the formation of Laves C14 and C15 phases. The Laves phase formation was attributed to micelle swelling and increased polydispersity driven by thermal caramelization of the sugar blocks. This work demonstrates that physical blending, combined with thermal modulation, enables access to the full spectrum of FK phases reported in block copolymers. These findings establish sugar-based BCOs as versatile and sustainable platforms for engineering soft-matter lattices through simple processing strategies.

## Graphical abstract

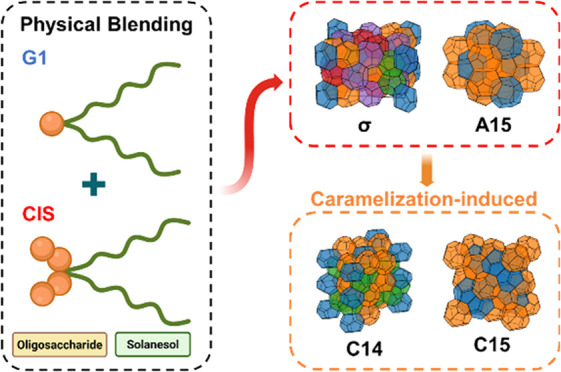

## Introduction

Glycolipids, a class of amphiphilic molecules with saccharide headgroups and hydrophobic tails, play essential roles in biological processes, including cell recognition, membrane stabilization, and signaling.^1–4^ Their biocompatibility, renewable nature, and ability to self-assemble into well-defined nanostructures have also made them promising candidates for pharmaceutical, biomedical, environmental, and nanotechnology applications.

An appealing characteristic of glycolipids is their ability to self-assemble into well-defined structures in both aqueous and melt states. In water, glycolipids form various nanostructured aggregates, including micelles, vesicles, and bicontinuous phases, depending on their molecular architecture and concentration.^5,6^ This self-assembly behavior arises from their amphiphilic nature, where the hydrophilic saccharide groups interact favorably with water, while hydrophobic tails drive phase separation to avoid water contact. In the melt state, the polar-nonpolar repulsion in glycolipids induces microphase separation, forming domain structures that closely resemble those found in block copolymers (BCPs).^7–9^ These thermotropic mesophases have also attracted significant interest due to their potential applications.

Inspired by the chemical structure of glycolipids, sugar-based block co-oligomers (BCOs) have been synthesized by covalently linking oligosaccharides with either synthetic or naturally occurring nonpolar blocks.^10–15^ This design strategy mimics the amphiphilicity of glycolipids while offering greater molecular tunability for expanding the self-assembled structure. In particular, sugar-based BCOs benefit from the strong hydrogen bonding and low conformational entropy of the oligosaccharide blocks, which enhance the interblock segregation and thus facilitate microphase separation in the oligomeric regime. This capability enables the formation of thermally stable nanostructures with ultrasmall feature sizes, making sugar-based BCOs highly attractive for nanoscale material design.^14,16–26^

By tuning their molecular architecture and composition, sugar-based BCOs have been successfully engineered to self-assemble into intricate morphologies, including bicontinuous double gyroid and hexagonal perforated layers.^9,15,24,26–28^ When the sugar block occupies a significantly smaller volume fraction relative to the nonpolar block, the system preferentially forms spherical micelles that pack into ordered lattices in the melt state.^11,15,21^ While body-centered cubic (BCC) packing is typically observed in sphere-forming BCP melts,^29,30^ sugar-based BCOs exhibit a distinct propensity for more complex packing arrangements, notably Frank–Kasper (FK) phases.^31–34^ Indeed, FK phases often replace BCC as the preferred lattice in sugar-based BCOs.

The first evidence of FK phase in a sugar-based BCO melt was reported by Sita et al. for cellobiose-*block*-atactic poly­(4-methyl-1-pentene) (CB-*b*-aPMP) which showed a rapid thermotropic transition from a hexagonally packed cylinder (HEX) phase to an exceptionally stable A15 phase.^31^ More recently, we demonstrated that an AB_2_ star-shaped BCO system, Glc_n_-(Sol)_2_, composed of an oligosaccharide block (Glc_n_, where Glc represents the glucose monomer and *n* is the degree of polymerization) and two solanesol (Sol) blocks (each Sol being a naturally occurring long-chain terpenoid comprising nine isoprene units with all-trans C=C double bonds), exhibits a systematically tunable sequence of micelle packing lattices.^34^ By increasing the length of the oligosaccharide block with single-monomer precision, the system underwent a phase transition from dodecagonal quasicrystal (DDQC)/BCC (*n* = 1) to FK σ (*n* = 2) to FK A15 (*n* = 3). Furthermore, stepwise cooling from the σ phase in Glc_2_-(Sol)_2_ induced its transformation to Laves C15 and C14 phases, demonstrating that this BCO system can access the full spectrum of FK phases previously observed in BCPs. The strong tendency of sugar-based BCOs to stabilize FK phases was attributed to their high-χ/low-*N* characteristics (with χ and *N* being the Flory–Huggins interaction parameter and degree of polymerization, respectively), which produced small micelles with significant interfacial free energy due to pronounced intermicellar contacts and core–corona repulsion. Under these conditions, the polyhedral deformation of micelles for space filling in FK phases reduces the total interfacial area compared to that in BCC lattice, thereby minimizing the overall interfacial free energy.^34–36^ These results highlight sugar-based BCOs as effective molecular design platforms for programming complex micelle packing in soft matter.

Despite the promising self-assembly behavior of sugar-based BCOs, synthesizing these materials with the precise conformational or architectural asymmetry necessary for accessing complex spherical phases remains a significant challenge. A practical strategy to overcome these synthetic difficulties is physical blending, where two or more distinct BCOs are combined to modulate their collective phase behavior. This approach offers a means to control the micelle size, size distribution, and interfacial curvature, which may in turn enhance the thermodynamic stability of FK phases. Indeed, physical blending has been theoretically predicted and experimentally validated as an effective method for stabilizing FK phases in the BCPs composed of two coil blocks.^36–42^ In such systems, the binary blends of BCPs or BCP/homopolymer blends can induce the formation of FK phases, including σ, A15, and Laves C14/C15.^36–40,43–51^

While blending has proven to be an effective strategy for inducing FK phases, its application to sugar-based BCOs remains relatively unexplored in the context of FK phase formation. Lachmayr and Sita demonstrated that the phase window of CB-*b*-aPMP could be broadened to accommodate both A15 and σ phases by incorporating small amounts of α-tocopherol as a phase modulator.^32^ Building on this approach, subsequent studies achieved a stable A15 phase at ambient temperature and observed a DDQC → A15 → σ phase transition with increasing temperature by blending monosaccharide- and disaccharide-polyolefin conjugates with α-tocopherol.^33^ These findings demonstrate physical blending with a foreign selective solvent as a viable alternative to precise molecular design for directing self-assembly into FK phases in sugar-based BCOs.

In this study, we extend our previous investigations of discrete oligosaccharide–solanesol BCOs to demonstrate that a single binary blend system, composed of two such BCOs, was capable of forming σ, A15, and Laves phases, collectively covering the full spectrum of FK phases previously identified in coil–coil BCP and sugar-based BCO systems. Specifically, we examine the phase behavior of the blends comprising glucose-(solanesol)_2_ (Glc_1_-(Sol)_2_) with an AB_2_ architecture (denoted as G1) and 1,3-(maltose)_2_-(solanesol)_2_ (1,3-(Glc_2_)_2_-(Sol)_2_) with an A_2_B_2_ architecture (denoted as CIS). According to our earlier work, neat G1 formed DDQC and BCC phases,^34^ while CIS formed lamellar (LAM) and hexagonally perforated layer (HPL) morphologies owing to higher sugar composition.^27^

Notably, although neither neat component independently formed FK phases, the micelles developed in their blends exhibited FK packing accompanied by complex phase evolution pathways. We found that G1-rich blends preferentially stabilized FK σ and A15 phases, while thermal processing drove the formation of Laves phases, attributed to significant micelle enlargement induced by sugar block caramelization at elevated temperatures. The FK phases observed in the blends closely mirrored those obtained from the discrete BCOs with precisely defined oligosaccharide lengths.^34^ However, blending offers practical advantages: it simplifies sample preparation and furthermore enables continuous tuning of the sugar block composition, in contrast to the compositionally discrete nature of neat BCOs, thereby providing enhanced control over a self-assembled structure.

## Experimental Section

### Materials and Sample Preparation

The monodisperse Glc_1_-(Sol)_2_ and 1,3-(Glc_2_)_2_-(Sol)_2_ were synthesized by click reaction, and the detailed procedures have been described elsewhere.^15^ Table 1 shows the chemical structures and molecular characteristics of the two BCOs. The blends of G1 and CIS were prepared by solvent casting. First, each BCO was dissolved in tetrahydrofuran (THF) at room temperature (ca. 1.0 wt %) and stirred in a sealed vial for at least 24 h. The two solutions were then combined in another sealed vial and stirred for at least 12 h to ensure complete mixing. The resulting mixture was poured into a Petri dish and allowed to evaporate at ambient temperature over 48 h. Finally, the sample was placed in a vacuum oven and dried at 30 °C for 3 h to remove any residual solvent. The dried samples were then used for SAXS experiments without further heat treatment. The blend composition is denoted as *W*_G1_/*W*_CIS_, where *W*_G1_ and *W*_CIS_ represent the weight percentages of G1 and CIS, respectively. An alternative expression of the blend composition is the overall sugar weight fraction, *w*_Glc_, and volume fraction, *f*_Glc_. The values of *W*_G1_/*W*_CIS_, *w*_Glc_, and *f*_Glc_ for the prepared blends are summarized in Table S1 of the Supporting Information (SI).

**Table 1:**
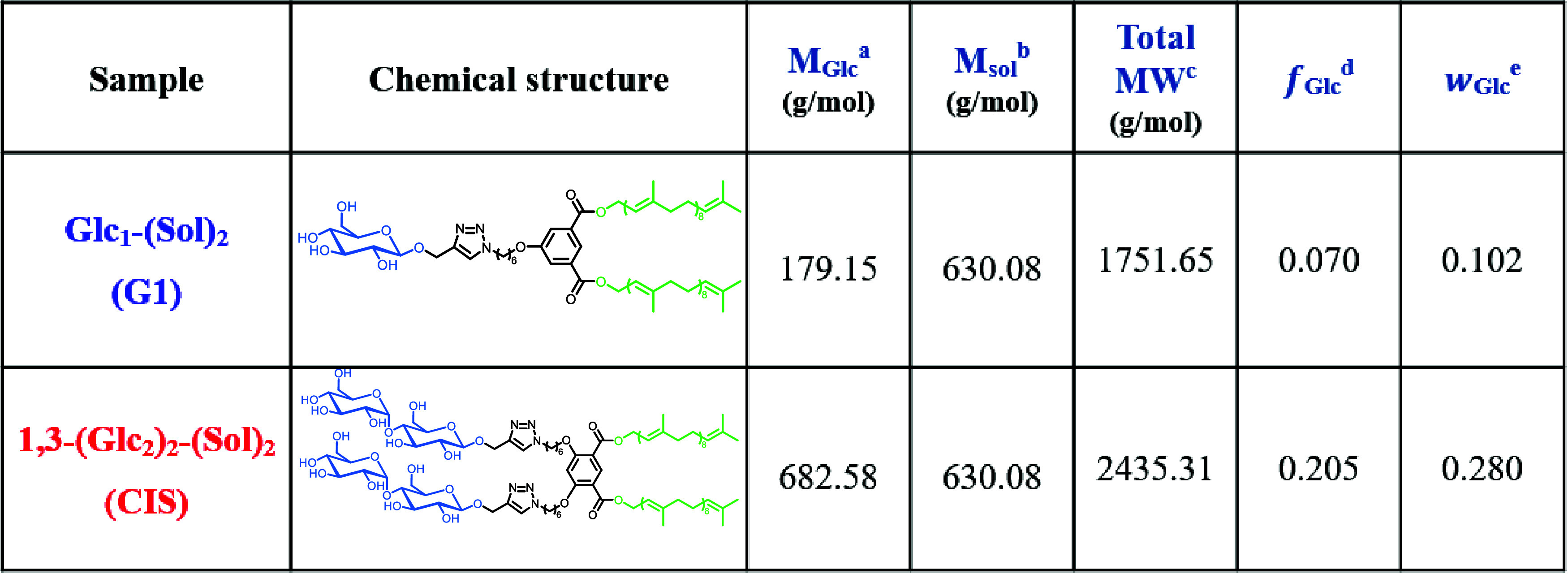
Molecular Characteristics of the BCO Samples Studied

a Molecular weight of the Glc_n_ block (marked with blue color).

b Molecular weight of one Sol block (marked with green color).

c Total molecular weight.

d Volume fraction of oligosaccharide block determined using the mass density values of 1.36 and 0.90 g/cm^3^ for the sugar block and the nonpolar component, including Sol and the linker, respectively.

e Weight fraction of oligosaccharide block.

### Small Angle X-ray Scattering (SAXS) Measurement

SAXS measurements were conducted at the TLS 23A1 SWAXS, TPS 13A BioSAXS, and TPS 25A1 coherent X-ray scattering beamlines at the National Synchrotron Radiation Research Center (NSRRC) located in Hsinchu, Taiwan. Monochromatic radiation with an energy of 15 keV was used, and the 2D scattering patterns were recorded with a PILATUS 1 M detector at TLS 23A1, a Dectrix EIGER X9M detector at TPS 13A, and an EIGER 16 M detector at TPS 25A1. The instrument covered the modulus of the scattering vector *q* of approximately 0.04 to 4 nm^–1^, where *q* is defined as *q* = 4π/λ sin­(θ/2), with λ and θ being the X-ray wavelength and the scattering angle, respectively.

For the temperature-dependent SAXS measurements, the sample was heated or cooled in 10 °C increments, with a 5 min isothermal hold at each temperature set point. The temperature ramp rate between set points was maintained at approximately 5 °C/min, yielding an average effective ramp rate of ∼1.43 °C/min over the full thermal cycle. The data acquisition times were 30 s at TLS 23A1, 1.0 s at TPS 13A, and 0.3 s at TPS 25A beamlines. The resulting 2D scattering patterns were radially averaged to generate 1D intensity profiles, which were subsequently corrected by subtracting background scattering.

### Size Exclusion Chromatography (SEC) Measurement

SEC measurements were conducted on the CIS to evaluate the effect of thermal treatment on its molecular weight. The thermal treatment was carried out using a NETZSCH DSC 200 F3Maia, differential scanning calorimeter (DSC), in which the sample was heated from 30 °C to target temperatures of 150, 170, 190, and 210 °C in 10 °C increments, with a 5 min isothermal hold at each set point, following the same protocol used in SAXS experiments. After each thermal cycle, the treated material was dissolved in THF and filtered through a 0.20 μm polytetrafluoroethylene (PTFE) syringe filter to remove any insoluble residues. A 100 μL aliquot was then injected into an Agilent Technologies 1260 Quaternary Pump VL (Model G1311C) gel permeation chromatography (GPC) system using THF as the mobile phase at a flow rate of 1.0 mL/min, with polystyrene as the calibration standard and an Agilent PLgel MIXED-D GPC column (cat. PL1110-6100). Shifts in the elution time were monitored to assess molecular weight changes induced by thermal treatment.

## Results and Discussion

### Equilibrium Phase Behavior of G1/CIS Blends

In our previous temperature-dependent SAXS study, G1, with a sugar weight fraction of *w*_Glc_ = 0.102, was found to exhibit a metastable liquid-like packing (LLP) phase in the as-prepared state.^34^ In this phase, micelles consist of an oligosaccharide core surrounded by a Sol corona packed with only short-range order. Upon heating, the system evolved from the LLP phase into an DDQC structure. The DDQC was hypothesized to be a metastable intermediate, potentially serving as a precursor to the σ phase.^37,52–54^ With a further temperature increase, the DDQC transformed into a BCC phase.

For neat CIS with *w*_Glc_ = 0.280, a LAM structure was initially observed. Upon heating, it transitioned to an HPL phase. The HPL structure was characterized by an ABCABC··· stacking sequence of the perforated layers, belonging to the *R*3̅*m* space group of the rhombohedral lattice.^27,55^

The phase behavior of the G1/CIS blend was also investigated by using temperature-resolved SAXS experiments under a dynamic heating ramp. The two BCO constituents share the same nonpolar Sol block but differ in the number of glucose units in the sugar block and their molecular architecture. Figure 1 presents the SAXS profiles of G1/CIS blends with 90/10 (*w*_Glc_ = 0.120) and 80/20 (*w*_Glc_ = 0.138) compositions during heating. For the 90/10 blend (Figure 1a), the SAXS curves below 50 °C showed a broad primary peak at 1.03 nm^–1^ and a weak shoulder near 1.75 nm^–1^, characteristic of an LLP phase.

**Figure 1:**
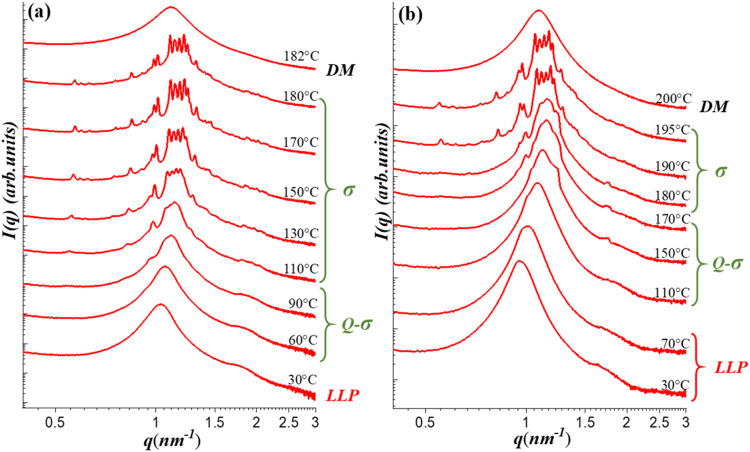
Temperature-dependent SAXS profiles of (a) G1/CIS 90/10 and (b) G1/CIS 80/20 blends at selected temperatures during the heating process from the as-prepared state. The samples were heated in 10 °C intervals at a ramp rate of approximately 5 °C/min. At each designated temperature, the sample was annealed for 5 min before SAXS data acquisition. The structures formed over the specific temperature ranges are indicated in the figure. The scattering peaks corresponding to the σ phase are satisfactorily indexed to the *P*4_2_/*mnm* space group of a tetragonal unit cell, as detailed in Figure S1 of the Supporting Information.

As the temperature increased to 60–90 °C, a weak peak emerged at 0.93 nm^–1^, indicating the development of an intermediate-range order. Upon further heating, this intermediate structure evolved into a well-defined FK σ phase, as evidenced by the emergence of multiple sharp diffraction peaks. These peaks were indexed to the *P*4_2_/*mnm* space group corresponding to a tetragonal unit cell with the lattice parameters *a* = 23.42 nm and *c* = 12.37 nm (*c*/*a* = 0.528), as shown in Figure S1 of the SI. This result confirms the successful induction of the FK σ phase within the blend. The intermediate structure observed between 60 and 90 °C is herein referred to as a “quasi-σ (Q-σ)” phase,^49^ in which the emerging peak at 0.93 nm^–1^ was attributed to layered ordering of the micelles^49,52,54^ while lateral order remained absent or poorly developed. At 182 °C, the SAXS pattern transformed into a broad featureless halo, indicative of a disordered micelle (DM) phase and marking the occurrence of an order–disorder transition (ODT).

Similarly, for the G1/CIS 80/20 blend, as shown in Figure 1b, the LLP phase was observed at low temperatures and gradually transformed into the σ phase upon heating, followed by the occurrence of ODT. In contrast, our previous study on neat G1 revealed a different phase transition pathway under the same thermal protocol: the LLP phase transformed into a DDQC upon heating, which then evolved into a BCC phase at higher temperatures without indication of σ phase formation.

Notably, in the blends, the σ phase emerged directly from the LLP phase, and no DDQC phase was detected. This transformation pathway persisted even with CIS content increased up to 40 wt %. These differences suggest two possible interpretations. One is that the LLP-to-σ transition in neat G1 is hindered by a high activation barrier, which is substantially lowered upon CIS incorporation, allowing the σ phase to form directly in the blends. The other possibility is that the DDQC observed in neat G1 is not merely a transient or metastable state but rather represents a thermodynamically stable structure.

While these interpretations align with our experimental observations, we acknowledge that the current data are insufficient to definitively assess the thermodynamic stability of the DDQC relative to that of the σ phase. Further theoretical and computational investigations tailored to the sugar-based BCO systems are required to elucidate the free energy landscape governing these phases and to determine whether the DDQC represents a true equilibrium structure or a metastable intermediate that precedes the formation of the σ phase.

Figure 2 shows the temperature-dependent variation of the average micelle volume ⟨*V*_m_⟩ within the σ phase formed in the G1/CIS 90/10, 80/20, and 70/30 blends. Here, ⟨*V*_m_⟩ is defined as *V*_uc_/*N*_p_, where *V*_uc_ is the volume of the tetragonal unit cell (given by *a* ^*2*^ *c*) and *N*_p_ = 30 represents the number of micelles per unit cell in the σ phase. The lattice parameters for the 70/30 blend were determined from the temperature-dependent SAXS profiles shown in Figure S2 of the SI. At any given temperature, ⟨*V*_m_⟩ increased with CIS content, with the 70/30 blend exhibiting the largest micelle size, followed by the 80/20 and 90/10 blends. This trend indicates that the micelles contained both BCO components. Specifically, the inclusion of the longer disaccharide blocks from CIS into the micelle core, originally composed of the Glc_1_ block from G1, resulted in core enlargement and, consequently, an increase in the micelle size.

**Figure 2:**
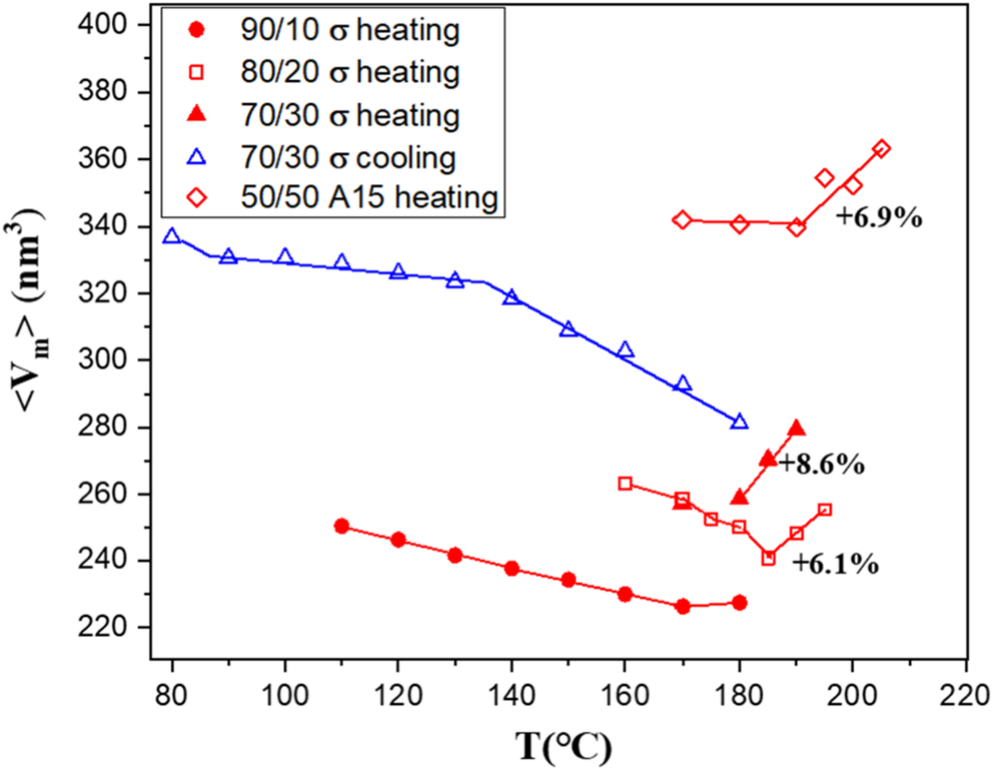
Temperature-dependent variation of the average micelle volume ⟨*V*_m_⟩ within the spherical phases formed in the G1/CIS blends. The micelle volume at a given temperature increased with increasing CIS composition, showing that CIS and G1 molecules are well mixed within individual micelles without evidence of macrophase separation. A noticeable micelle expansion is observed at elevated temperatures during the heating process, with the percentage of volume increase labeled in the figure. This anomalous growth is attributed to partial caramelization of the sugar blocks occurring at higher temperatures.

For the 90/10 blend, ⟨*V*_m_⟩ gradually decreased with increasing temperature from 110  to 170 °C, consistent with the expected reduction in χ parameter at elevated temperatures. However, a slight increase in ⟨*V*_m_⟩ was observed between 170  and 180 °C. This anomalous behavior was even more pronounced in the 80/20 and 70/30 blends, where ⟨*V*_m_⟩ of the σ phase exhibited a clear thermally induced expansion. In the 80/20 blend, micelle swelling became evident above 185 °C, resulting in an approximate 6.1% increase in ⟨*V*_m_⟩ between 185  and 195 °C. The 70/30 blend showed an earlier onset of expansion, with micelle growth initiating above 170 °C and reaching an approximate 8.6% increase by 190 °C.

The unusual micelle swelling at elevated temperatures was attributed to the partial caramelization of the sugar blocks which occurred above ca. 170 °C. Caramelization involves dehydration, structural rearrangement, and potential cross-linking, as reported in several studies^11,17,56–59^ These chemical changes may alter the average molecular weight, the polydispersity, and even the χ parameter of the BCOs, resulting in an overall expansion of the micelle core. Evidence supporting the occurrence of caramelization is displayed in Figure S3 of the SI, showing the evolution of surface coloration of the neat CIS sample from its pristine white state with increasing annealing temperature. After annealing at 150 °C, a faint yellow tint emerged at the periphery. Annealing at 170 °C produced a more uniform and intensified yellow coloration across the entire surface. Further heating to 190 °C yielded a pronounced amber tone indicative of advanced chromophore formation. Finally, at 210 °C, the sample exhibited a deep brown coloration consistent with extensive caramelization. Further analytical evidence of caramelization to be presented in the following section corroborates these visual changes.

When the CIS composition was increased to 50 wt % (*w*_Glc_ = 0.191), HEX became the stable structure below 150 °C, as evidenced by the SAXS peak position ratios of 1:3^1/2^: 4^1/2^ (Figure 3a). Upon heating to 160 °C, the HEX scattering pattern was replaced by the one consistent with the FK A15 phase, as confirmed by the peak indexing using the *Pm*3̅*n* space group of a cubic unit cell with the lattice parameter *a* = 14.0 nm (see Figure S4 of SI). In the A15 structure, the cubic unit cell contains eight particles, with two occupying Z12 coordination sites and six occupying Z14 sites.^31,60^ Notably, the transition from HEX to A15 occurred directly and rapidly, without passing through an LLP intermediate, mirroring the behavior observed in both CB-*b*-aPMP reported by Sita et al.^31^ and Glc_3_-(Sol)_2_.^34^ The A15 phase remained stable up to 205 °C, beyond which an ODT occurred.

**Figure 3:**
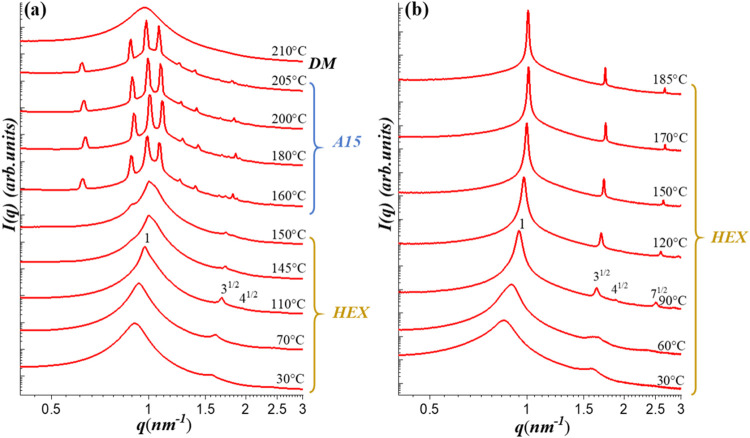
Temperature-dependent SAXS profiles of (a) G1/CIS 50/50 and (b) G1/CIS 25/75 blends at selected temperatures during the heating process from the as-prepared state. The samples were heated in 10 °C intervals at a ramp rate of approximately 5 °C/min. At each designated temperature, the sample was annealed for 5 min before SAXS data acquisition. The structures formed over the specific temperature ranges are indicated in the figure. The scattering peaks corresponding to the A15 phase are satisfactorily indexed to the *Pm*3̅*n* space group of a cubic unit cell, as detailed in Figure S4 of the Supporting Information.

The temperature variation of ⟨*V*_m_⟩ in the A15 phase of the 50/50 blend was also displayed in Figure 2. The micelle volume was significantly larger than that of the 70/30 blend, confirming that CIS and G1 molecules were well mixed within the individual micelles without macrophase separation. The micelle volume decreased progressively with increasing temperature up to 190 °C. However, between 190  and 205 °C, caramelization-induced structural changes led to an expansion of the micelles by approximately 6.9%. This nonmonotonic thermal response reflects the complex interplay between interblock interactions and thermally induced chemical modifications of the sugar block, both of which critically influenced the phase behavior of the sugar-based BCO.

The composition window for the formation of the A15 phase was found to span from *w*_Glc_ = 0.191 to 0.209. Beyond this range, further increases in the CIS content suppressed the formation of spherical phases. As shown in Figure 3b for the G1/CIS 25/75 blend with *w*_Glc_ = 0.236, the system remained in the HEX phase throughout the entire heating process.

The temperature-dependent SAXS experiments revealed the phase evolution of the G1/CIS blends during heating, as summarized in the morphological diagram in Figure 4. In their neat states, G1 and CIS respectively formed DDQC/BCC and LAM/HPL phases. However, simple physical blending of these BCOs gave rise to emergent behavior, notably the appearance of FK phases, not present in either component alone. Even a small addition of CIS was sufficient to induce the formation of σ phase in the blend. As the CIS content increased further, the σ phase was progressively destabilized, and at elevated temperatures, it transitioned into the A15 phase. When the CIS composition exceeded 60 wt %, the system remained entirely in the HEX phase throughout the heating process.

**Figure 4:**
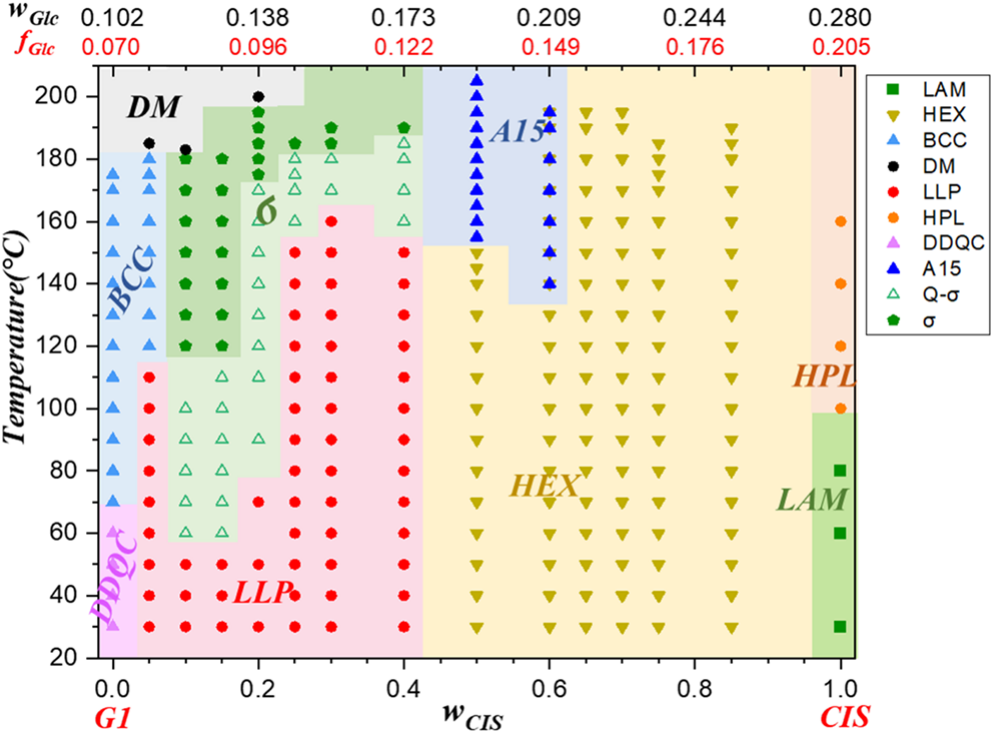
Morphological diagram of G1/CIS blends determined from the SAXS results collected in the heating process.

At higher temperatures (e.g., 170 °C), the observed phase progression with increasing CIS (and thus the overall sugar content) followed a clear sequence: BCC → σ → A15 → HEX. This lyotropic transition mirrors the phase sequence observed in neat Glc_n_-(Sol)_2_ BCOs with varying oligosaccharide length, where increasing the number of glucose units from one to two to three shifted the micelle packing from BCC to σ to A15.^34^ The results presented here demonstrate that the same FK phases can be conveniently accessed via a simpler blending strategy, bypassing the need for synthesizing discrete BCOs with specific sugar block lengths.

Notably, the observed lyotropic behavior is also qualitatively consistent with predictions from self-consistent field theory (SCFT)^41,42,60,61^ to experimental findings for coil–coil BCPs.^60,62^ This agreement is particularly interesting given that the sugar-based BCOs deviated significantly from the Gaussian chain statistics typically assumed in SCFT, owing to the conformational rigidity of the saccharide segments and the oligomeric nature of the BCO.

According to the morphological diagram, the formation of the σ phase upon the addition of CIS persisted up to a CIS content of 40 wt %. As a result, the σ phase was accessible over a sugar composition range of approximately *w*_Glc_ = 0.120 to 0.173. In contrast, for neat Glc_n_-(Sol)_2_ BCOs, the σ phase was only observed in Glc_2_-(Sol)_2_ with *w*_Glc_ = 0.177 at high temperatures (>190 °C), while further increasing the number of glucose units to three led to the formation of the A15 phase in Glc_3_-(Sol)_2_. This limitation stems from the discrete nature of the oligosaccharide block length, which results in a stepwise variation in *w*_Glc_ with relatively large intervals (Δ*w*_Glc_ ≈ 0.050–0.075). In contrast, the blending strategy employed in this study allows for virtually continuous tuning of the sugar composition, thereby facilitating broader and more precise access to the σ phase. This continuous adjustability represents an advantage over the compositionally discrete nature of neat BCO systems, effectively expanding the accessible phase space and enhancing the structural tunability.

It is worth noting that the A15 phase was observed at elevated temperatures in the Glc_3_-(Sol)_2_ BCO, which has a sugar weight fraction of *w*_Glc_ = 0.231.^34^ In contrast, the G1/CIS 30/70 blend, which has a similar overall sugar composition (*w*_Glc_ = 0.227), formed an HEX morphology. This difference can be attributed to the larger micelle core size in the blend compared to that in the neat Glc_3_-based BCO. In the G1 molecule, which consists of a single glucose unit attached to two Sol blocks, the small cross-sectional area of the Glc unit at the core–corona interface results in significant crowding of the Sol chains. This crowding induces the micelle core to adopt a higher interfacial curvature to relieve chain stretching. Additionally, the hydrogen bonding interactions between short Glc blocks are relatively weak and insufficient to offset the conformational entropic penalty associated with chain crowding, further promoting high-curvature micelle formation.

In contrast, CIS molecules, composed of two Glc_2_ blocks and two Sol chains, provide a bulkier sugar headgroup that increases the available interfacial area, thereby reducing crowding of the Sol blocks. Moreover, the longer Glc_2_ blocks support stronger hydrogen bonding interactions, which favor micelle formation with larger core sizes and lower interfacial curvature to enhance these interactions. As a result, the incorporation of CIS into the micelle leads to a reduction in average interfacial curvature, and under the same overall sugar composition, the blend preferentially forms a less curved HEX morphology, unlike the neat Glc_3_-based BCO, which stabilizes the A15 phase.

### Generation of Laves Phase by Thermal Processing

The observed FK phases and phase transition sequence were largely consistent with those reported in the experimental and SCFT studies of the binary blends of coil–coil BCPs.^36–42,63^ Under the experimental conditions and blend compositions employed in this study, we did not observe the formation of Laves phases, suggesting that such structures are likely not thermodynamically favored in the sugar-based BCO blend system.

In neat coil–coil BCP systems, Laves phases are typically metastable and were accessed through optimized thermal processing protocols.^35,64–66^ In contrast, blending strategies have been shown to stabilize these complex packing structures thermodynamically. For instance, incorporating homopolymers into micelle cores under dry-brush conditions can drive a sequential transformation from the σ phase to Laves C14, and eventually to C15 phase, with increasing homopolymer loading.^46^ Likewise, Laves phases have been reported across various compositional regimes in the blends of two coil–coil BCPs, A_1_B_1_ and A_2_B_2_, with differing chain lengths and block compositions.^37,40–42,63^ The SCFT calculations conducted by Shi et al. suggest that realizing the Laves phases in such binary blends requires the establishment of a core–shell micelle architecture, wherein the additive A_2_B_2_ copolymer possesses a high A-block volume fraction and a molecular weight equal to or greater than that of the host A_1_B_1_ chains.^41,42^ This arrangement promotes differential segregation between the core and shell domains, which in turn enables fine control over the micelle size distribution and facilitates the stability of Laves phases.

In our previous study on the formation of FK phases in Glc_n_-(Sol)_2_ systems, we observed that cooling Glc_2_-(Sol)_2_ from 197 to 190 °C induced a transition from σ to Laves phase.^34^ This transformation in micelle packing symmetry was accompanied by a substantial volume expansion of approximately 30%, indicating that the resulting larger micelle size favored the formation of the Laves lattice. The volume increase could arise from the caramelization of the oligosaccharide block at sufficiently high temperature, which was not noted in the previous work.^34^

In light of the previous findings, we conducted a thermal cycling experiment on the blends to investigate whether Laves phase formation could be induced via the treatment. The sample was first heated stepwise from the as-cast state to a temperature above the ODT temperature (*T*_ODT_), followed by stepwise cooling to 30 °C, and finally subjected to a second stepwise heating cycle.

Figure 1b demonstrates that the 80/20 blend in the first heating cycle exhibited the phase transition from LLP to Q-σ, and eventually to a well-ordered σ phase followed by the ODT. During the subsequent cooling cycle from the DM phase, as shown in Figure 5a, the micelles retained a liquid-like arrangement above 150 °C. As the temperature dropped below 150 °C, two scattering peaks appeared at 0.55 and 1.9 nm^–1^ and persisted down to 30 °C, indicating the development of an intermediate degree of micelle ordering. Upon immediate reheating, a well-defined set of peaks emerged, where the scattering profile gradually evolved into that characteristic of the Laves C14 phase, as shown in Figure 5b. Indexing of the observed peaks according to the *P*6_3_/*mmc* space group (see Figure S5 of SI) yielded the lattice parameters of *a* = 13.96 nm and *c* = 22.84 nm (*c/a* = 1.636) for the hexagonal unit cell of the C14 phase. It is noted that the weak peak initially observed at 0.55 nm^–1^ progressively sharpened and split into distinct reflections corresponding to the (100), (002), and (101) planes of the C14 phase. Therefore, the micelle arrangement observed below 150 °C during cooling and below 140 °C during the subsequent reheating cycle was referred to as the “quasi-Laves” (Q-Laves) phase, an intermediate state that preceded full transformation into the ordered Laves C14 lattice.

**Figure 5:**
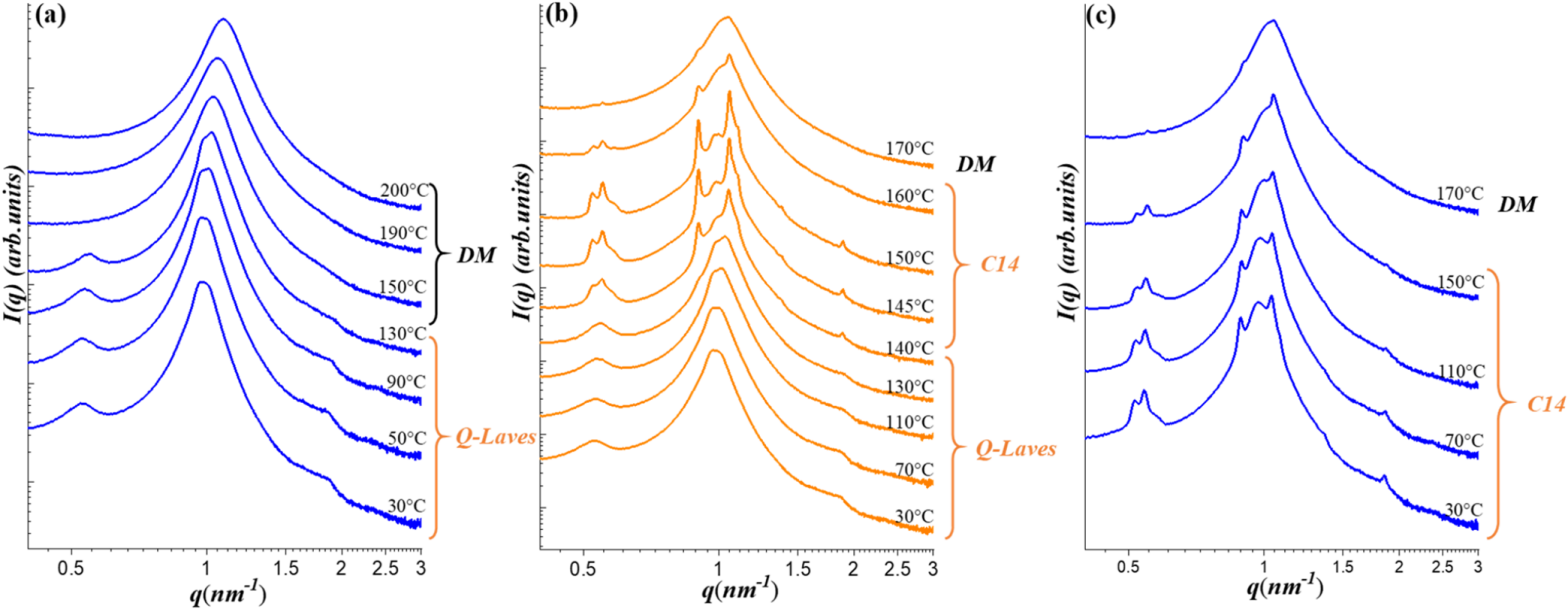
Temperature-dependent SAXS profiles of the G1/CIS 80/20 blend at selected temperatures during a thermal cycling experiment. The sample was initially heated stepwise from the as-prepared state to the disordered micelle (DM) phase (Figure 1b), followed by cooling, reheating, and a subsequent cooling cycle. Panels (a) and (b) show the SAXS profiles collected during the cooling and reheating processes, respectively. Panel (c) presents an additional cooling process following reheating to 170 °C, illustrating the reappearance of the C14 phase upon cooling from the DM state. The identified structural phases at various temperatures are labeled in the figure.

The appearance of the Q-Laves phase in the cooling cycle suggests the development of partial or short-range Laves-like ordering among micelles, which did not fully evolve into a long-range ordered structure at this stage. This behavior may be attributed to the presence of a kinetic barrier that hindered the formation of the fully developed Laves phase during cooling. The reorganization of micelles into the highly specific coordination required for Laves structures, particularly those with significant micelle size disparities such as C14 and C15, was kinetically suppressed at lower temperatures due to restricted mobilities of the chains and micelles. As a result, the system became kinetically trapped in a metastable, locally ordered state, i.e., the Q-Laves phase, where the micelles exhibited partial positional and orientational correlation consistent with Laves symmetry but lacked long-range coherence. In the second heating cycle, the thermal energy provided enabled the system to overcome the kinetic constraints, allowing micelles to reorganize and pack into the ordered C14 lattice.

The Laves phase formed in the second heating cycle transformed into the DM phase at 170 °C. Upon subsequent cooling to 150 °C (i.e., during the second cooling process), the C14 phase re-emerged, as shown in Figure 5c. This observation indicates that the Laves structure can persist or reform even after multiple thermal cycles. However, repeated thermal cycling was found to progressively lower the *T*_ODT_, likely due to subtle chemical changes, such as partial caramelization, that diminish the thermal stability of the ordered phase.

While the σ-forming 80/20 blend was shown to transform into the Laves C14 phase upon thermal cycling, we further examined whether a similar transition could occur in the A15-forming 50/50 blend. The SAXS profiles collected during the first heating cycle (see Figure 3a) indicated that this blend entered a DM phase at 210 °C. During the subsequent cooling cycle, as displayed in Figure 6a, the micelles retained a liquid-like packing above 150 °C. Upon further cooling to 130 °C, a weak scattering peak emerged at 0.48 nm^–1^. Notably, the SAXS profiles obtained between 130 and 30 °C closely resembled those of the Q-Laves phase previously identified in the 80/20 blend.

**Figure 6:**
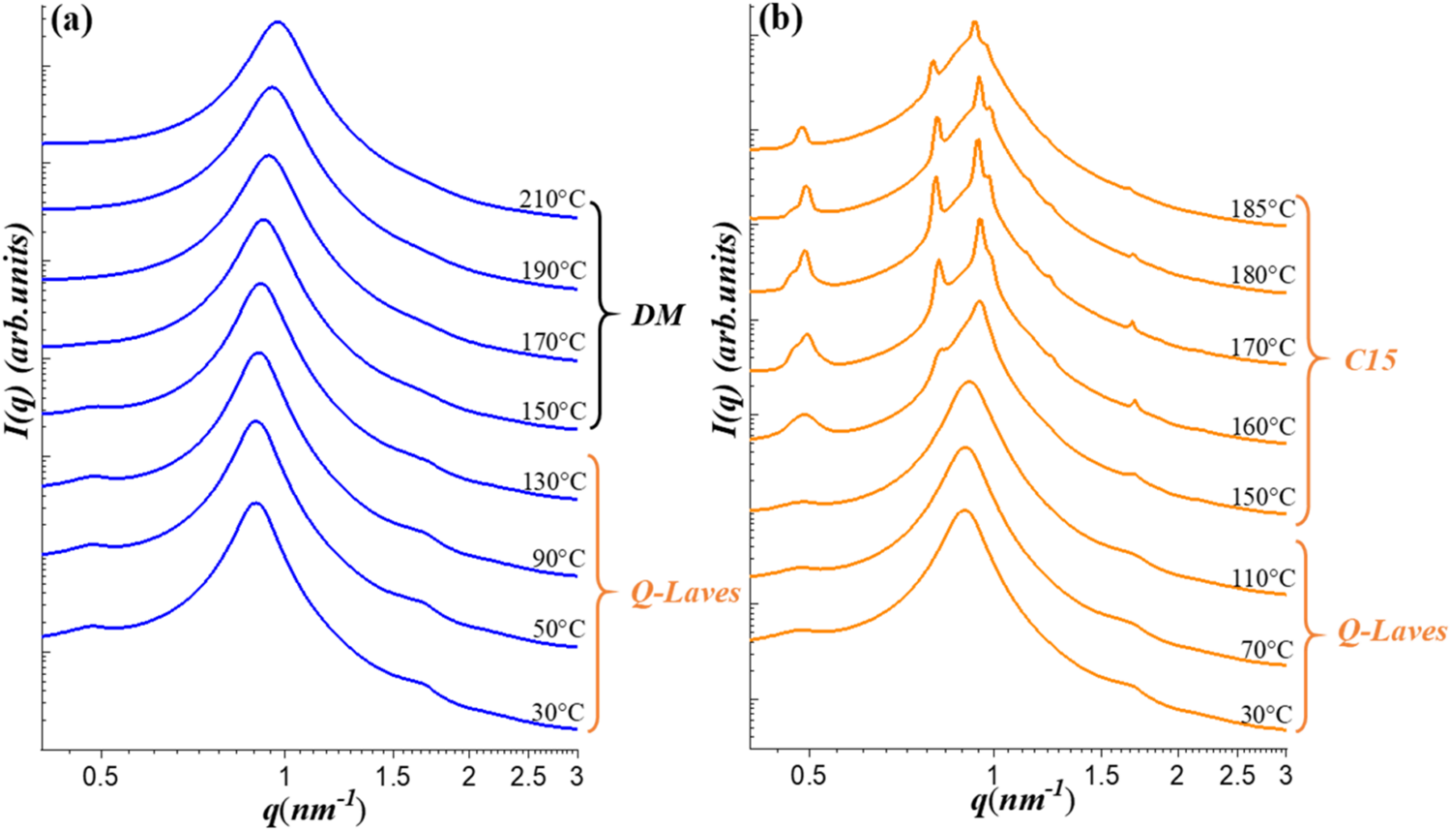
Temperature-dependent SAXS profiles of the G1/CIS 50/50 blend at selected temperatures during a thermal cycling experiment. The sample was initially heated stepwise from the as-prepared state to the disordered micelle (DM) phase (Figure 3a), followed by cooling and subsequent reheating. Panels (a) and (b) show the SAXS profiles collected during the cooling and reheating processes, respectively. The structural phases identified over specific temperature ranges are indicated in the figure. The Laves C15 phase was observed to emerge during the reheating process.

In the second heating cycle, the diffraction peaks associated with the Q-Laves phase gradually sharpened, and additional reflections emerged as the temperature increased, as demonstrated in Figure 6b. These features ultimately evolved into a well-resolved diffraction pattern characteristic of the Laves C15 phase. Indexing of the scattering peaks according to the *Fd*3̅*m* space group yielded a lattice parameter of *a* = 22.1 nm for the cubic C15 unit cell, as displayed in Figure S6 of the SI.

It is important to note that the formation of Laves phases is not guaranteed simply by heating to the DM state, followed by cooling. To evaluate the robustness of this thermal cycling strategy, we conducted an additional SAXS experiment on the 50/50 blend, this time with a slightly higher maximum temperature of 215 °C (see Figure S7 in SI). In this case, although the A15 phase initially formed and underwent an ODT upon heating, the scattering peaks became noticeably broader and shifted to lower *q* at 215 °C, indicating micelle swelling and increased structural disorder due to overheating. Upon subsequent cooling, no discernible signatures of the Q-Laves or Laves phase were observed, and the system remained disordered even after reheating. These findings highlight the critical importance of proper thermal control, where overheating beyond an optimal processing window can suppress the formation of the desired complex packing structures. The emergence of Laves phases in sugar-based systems is therefore sensitive to thermal history, emphasizing the need for carefully regulated annealing protocols.

Figure 7a displays the average micelle volume for the G1/CIS 80/20 blend during the first and second heating cycles, corresponding to the formation of the σ phase and the C14 phase, respectively. In the first heating cycle, the micelle volume in the σ phase increased by approximately 6.1% above 185 °C, likely due to caramelization effects. In the second heating cycle, the micelles in the C14 phase are substantially larger, about 28% greater than those in the σ phase found in the first cycle. A similar trend was observed in the 50/50 blend (see Figure 7b), where the C15 phase showed a micelle volume approximately 25% larger than that of the A15 phase.

**Figure 7:**
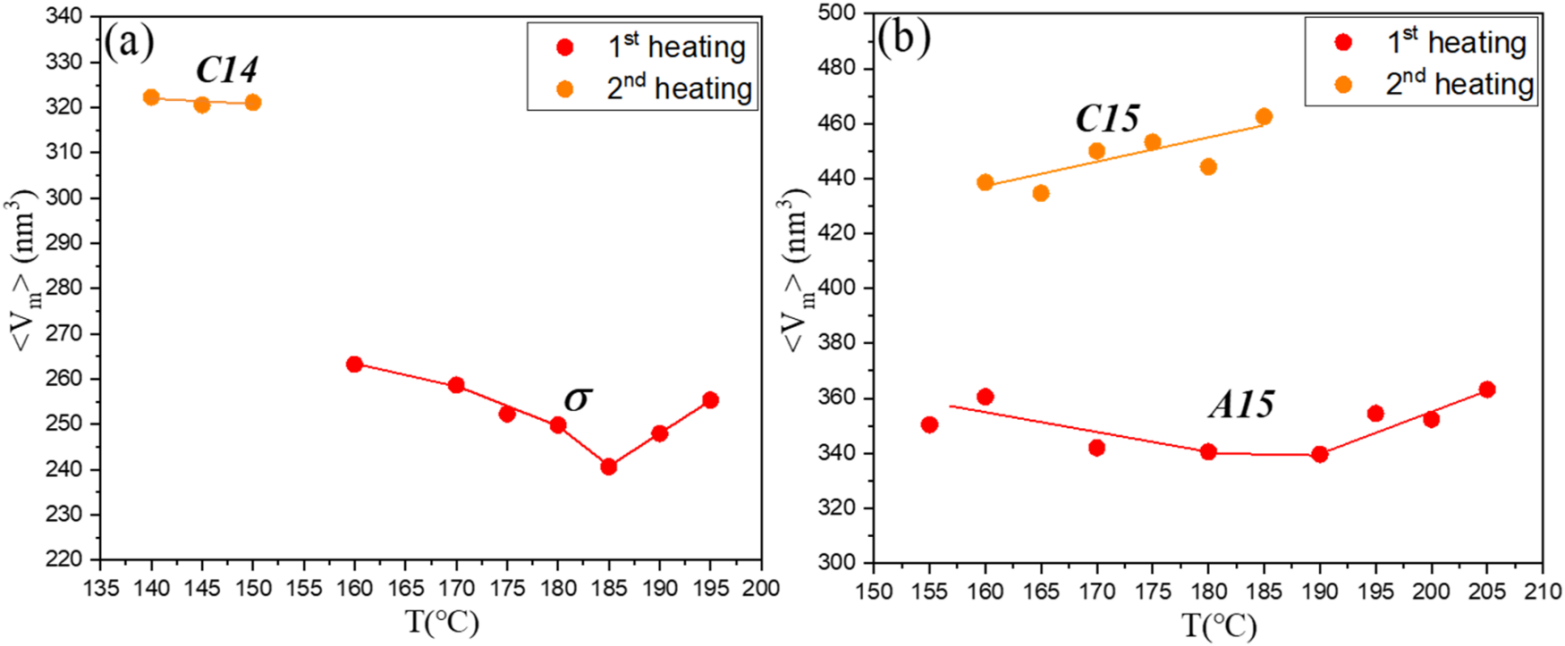
Temperature-dependent variation of the average micelle volume ⟨*V*_m_⟩ in G1/CIS (a) 80/20 and (b) 50/50 blends during the first and second heating cycles. In the second cycle, micelles forming the Laves phase exhibit substantially larger volumes compared to those in the σ and A15 phases observed during the first heating cycle.

To investigate whether the formation of the Laves phase could be attributed to caramelization of the oligosaccharide block, we deduced the onset of caramelization at elevated temperatures by performing SEC analysis on neat CIS, which was subjected to a stepwise heating protocol identical to the initial heating cycle of the temperature-dependent SAXS experiment. Specifically, the sample was annealed at each prescribed temperature for 5 min, with the temperature incremented in 10 °C intervals up to the target temperature *T*_a_. In this study, four samples were prepared by heating them to *T*_a_ values of 150, 170, 190, and 210 °C, respectively. Figure 8a displays the SEC elution curves of the thermally treated samples alongside those of the 30 °C reference. The elution profiles were clearly affected by *T*_a_; in particular, above 190 °C, the main peak shifted to shorter elution times and became significantly broader, indicating an increase in the average molecular weight and a rise in the polydispersity index (*Đ*), as illustrated in Figure 8b. Notably, annealing at 210 °C led to the emergence of a distinct shoulder around 29 min, suggesting the formation of low-molecular-weight (low-MW) species likely resulting from extensive caramelization of CIS. In this case, partial caramelization of the maltose blocks led to the formation of both low-MW species (e.g., hydroxymethylfurfural (HMF) and other dehydration products) and higher-MW co-oligomers through condensation and polymerization reactions.^56–59^

**Figure 8:**
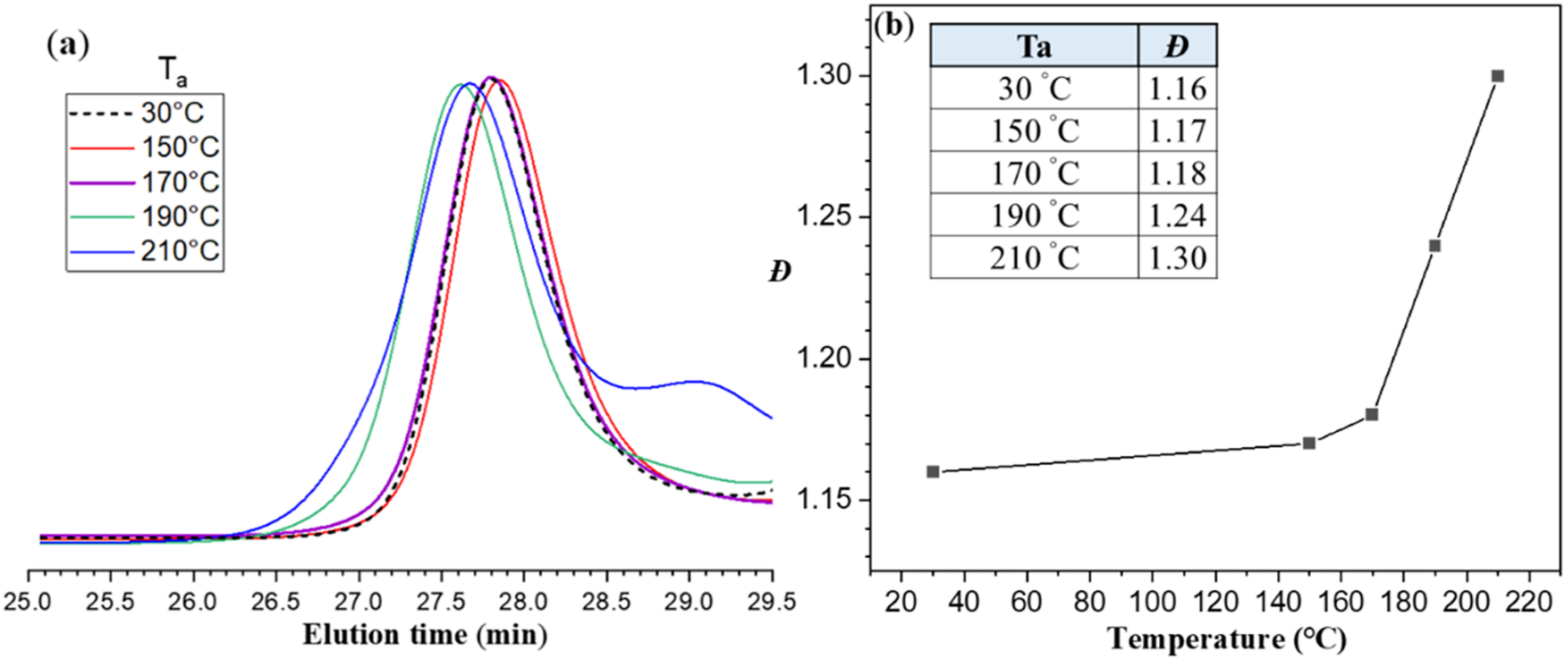
(a) SEC elution curves of thermally treated CIS samples compared to the 30 °C reference. The elution profiles are influenced by the annealing temperature (*T*_a_), with the main peak shifting to shorter elution times and broadening as *T*_a_ increases, indicating an increase in average molecular weight and polydispersity index (*Đ*), as shown in panel (b). Notably, annealing at 210 °C results in the appearance of a distinct shoulder at approximately 29 min, suggesting the formation of low-molecular-weight species likely generated through extensive caramelization of CIS.

The SEC analysis confirmed that caramelization of the oligosaccharide block in CIS occurred at elevated temperatures, particularly above 190 °C. This process produces both low- and higher-MW species, significantly broadening the molecular weight distribution of the BCO. It is likely that these species were effectively incorporated into the micelles and resulted in a net increase in the average micelle volume. Concurrently, the presence of larger, cross-linked fragments may also enhance micelle rigidity and effective volume. These combined effects lead to substantial micelle size polydispersity and an overall increase in volume asymmetry.

Such a broad volume distribution among micelles is particularly relevant to the formation of the Laves phase.^65–67^ Laves phase is known to accommodate particles with a significant size asymmetry (*V*_L_/*V*_S_ = 1.23 for the volume ratio of the largest to the smallest particles as compared to the value of approximately 1.16 in the σ and A15 phases)^38,47^ by arranging them into interpenetrating lattices with broadly different local environments. In this context, the coexistence of larger and smaller micelles within the system, promoted by both volume expansion and polydispersity induced by caramelization, created favorable conditions for Laves phase formation. Thus, the appearance of the Laves phase in the blends at elevated temperatures can be reasonably attributed to micelle size diversification arising from the occurrence of caramelization. The enhanced micelle size dispersity is evidenced by the broader scattering peak of the LLP phase from which the Q-Laves phase evolved, as observed in the SAXS profiles at 150–200 °C for 80/20 blend (Figures 5a and S8), in contrast to the narrower peak associated with the LLP phase at 30–110 °C that precedes σ-phase formation (Figures 1b and S8).

These findings are strongly supported by theoretical and experimental studies in the literature.^68,69^ Lai and Shi employed SCFT to show that increasing molecular weight polydispersity in conformationally asymmetric AB diblock copolymers systematically broadens the stability window of FK phases, including σ, A15, and Laves C14/C15, even making them true equilibrium structures when a Schulz-Zimm distribution is used.^69^ On the experimental side, Cheng and co-workers established volume heterogeneity as a unifying design principle for complex spherical packings. They first demonstrated that triphenylene-polyhedral oligomeric silsesquioxane (POSS) amphiphiles exhibit a thermally driven A15→Z transition once their micelle volume asymmetry exceeds a critical threshold dictated by core–shell segregation.^70^ Subsequently, they showed that precise geometric control of linker length and cage/core dimensions enables the formation of the FK Z phase.^71^ Further, they reported that assemblies of 5-fold symmetric molecular pentagons could access both the μ and a novel ϕ phase when broad micelle size and shape distributions arise.^72^ Most recently, they demonstrated that blending architecturally defined giant amphiphiles in controlled ratios allows fine-tuning of the mesoatom volume ratio *V*_L_/*V*_S_, enabling access to a wide array of soft-matter lattices, including BCC, FCC, σ, A15, Laves C14/C15, NaZn_13_/A_l_B_2_, and even NaCl-type superlattices.^73^

In light of these insights, we conclude that caramelization in sugar-based BCO serves as an effective, temperature-triggered strategy for modulating micelle volume dispersity and symmetry breaking, thereby promoting the formation of Laves phases from the previously stable σ or A15 phases. This approach highlights the broader potential of leveraging thermal reactivity to guide the emergence of complex spherical packing motifs in sugar-based BCOs. Further investigations are necessary to elucidate the detailed kinetic pathways underlying this phenomenon.

## Conclusions

In this study, we investigated the self-assembly behavior of the binary blends of chemically distinct sugar-based BCOs, G1 (Glc_1_-(Sol)_2_, AB_2_-type) and CIS (1,3-(Glc_2_)_2_-(Sol)_2_, A_2_B_2_-type), to explore the use of physical blending as a strategy to access complex micelle packing structures. While the neat components formed distinct morphologies: DDQC/BCC for G1 and LAM/HPL for CIS, their blends revealed a rich array of nanostructures, including the emergence of FK σ and A15 phases. Notably, we observed a lyotropic phase transition sequence of BCC→ σ → A15 → HEX with an increasing overall sugar content in the blends. This phase evolution is consistent with theoretical predictions for coil–coil BCPs, despite the conformational differences between sugar-based BCOs and flexible Gaussian coils.

Beyond the compositional tuning, we further demonstrated that thermal annealing drives the formation of even more complex packings, specifically the Laves C14 and C15 phases. SAXS and SEC analyses indicate that this transformation was closely linked to partial caramelization of the oligosaccharide blocks at elevated temperatures. Caramelization produced a mixture of low- and higher-MW species, which led to micelle swelling and increased size polydispersity. This dynamic enhancement of micelle volume asymmetry correlated with the stabilization of the Laves phases.

These results demonstrate that binary blending, coupled with controlled thermal treatment, provides a powerful and scalable method for tuning the micelle size distribution and accessing a broad spectrum of FK phases in sugar-based systems. Future work will focus on elucidating the detailed kinetics of phase transitions and extending this blending-based approach to other renewable molecular systems.

## Supplementary Material



## Notes

Compositions and the corresponding overall sugar weight fractions of the studied blends; SAXS profile of the G1/CIS 90/10 blend collected at 170 °C, indexed to the characteristic reflections of the Frank–Kasper σ phase; temperature-dependent SAXS profiles of G1/CIS 70/30 blends collected during heating and subsequent cooling process from the as-prepared state; visual evidence of thermally induced caramelization in the neat CIS after thermal annealing; SAXS profile of G1/CIS 50/50 blend collected at 190 °C, indexed to the characteristic reflections of the Frank–Kasper A15 phase; SAXS profile of the G1/CIS 80/20 blend collected during the reheating process to 150 °C, indexed to the characteristic reflections of the Laves C14 phase; SAXS profile of G1/CIS 50/50 blend collected during the reheating processes to 180 °C, indexed to the characteristic reflections of the Laves C15 phase; temperature-dependent SAXS profiles of G1/CIS 50/50 blends collected under a modified heating protocol; temperature dependence of the SAXS peak position (*q**) and full-width at half-maximum (fwhm) of the LLP phases formed in G1/CIS 80/20 blend (PDF)
