# Supplementary material for: Accessing Frank–Kasper Phases via Blending of Architecturally Distinct and Sustainable Sugar-Based Block Co-Oligomers
Source: Macromolecules. 2025 Aug 6;58(16):8686–97. doi: 10.1021/acs.macromol.5c01479 (PMC12392730; doi:10.1021/acs.macromol.5c01479)
Supplement: Supplementary file 1 [file ma5c01479_si_001.pdf]

Supporting Information

Accessing Frank-Kasper Phases via Blending of Architecturally Distinct and Sustainable Sugar-based Block Co-Oligomers

Yu-Hung Cheng[1], Ting-Wei Chang[1], Taiki Nishimura[2], Yi-Cheng Lai[3], Chun-Jen Su[3], Jane Wang[1], Takuya Isono[4]*, Toshifumi Satoh[4*], Hsin-Lung Chen[1]*

[1]Department of Chemical Engineering, National Tsing Hua University, Hsinchu 300043, Taiwan

[2]Graduate School of Chemical Sciences and Engineering, Hokkaido University, Sapporo 060-8628, Japan

[3] National Synchrotron Radiation Research Center, Hsinchu 300092, Taiwan

[4]Faculty of Engineering, Hokkaido University, Sapporo 060-8628, Japan

Corresponding Author

Hsin-Lung Chen: hlchen@che.nthu.edu.tw

Takuya Isono: isono.t@eng.hokudai.ac.jp

Toshifumi Satoh: satoh@eng.hokudai.ac.jp

1. Compositions, the corresponding overall sugar weight fractions, and volume fractions of the studied blends.

Table S1. Blend compositions (W_{G1}/W_{CIS}) and corresponding overall sugar weight fractions (w_{Glc}) and volume fractions (f_{Glc}). W_{G1} and W_{CIS} denote the weight percentages of G1 and CIS in each blend, respectively. The blend composition is also expressed in terms of the total sugar content, represented by w_{Glc} and f_{Glc}

W_{G1}/W_{CIS}	w_{Glc}	f_{Glc}
100/0	0.102	0.070
95/5	0.111	0.076
90/10	0.120	0.083
85/15	0.129	0.089
80/20	0.138	0.096
75/25	0.147	0.102
70/30	0.155	0.108
60/40	0.173	0.122
50/50	0.191	0.135
40/60	0.209	0.149
35/65	0.218	0.156
30/70	0.227	0.163
25/75	0.236	0.170
15/85	0.253	0.183
0/100	0.280	0.205

2. SAXS profile of the G1/CIS 90/10 blend collected at 170 °C, indexed to the characteristic reflections of the Frank–Kasper σ phase

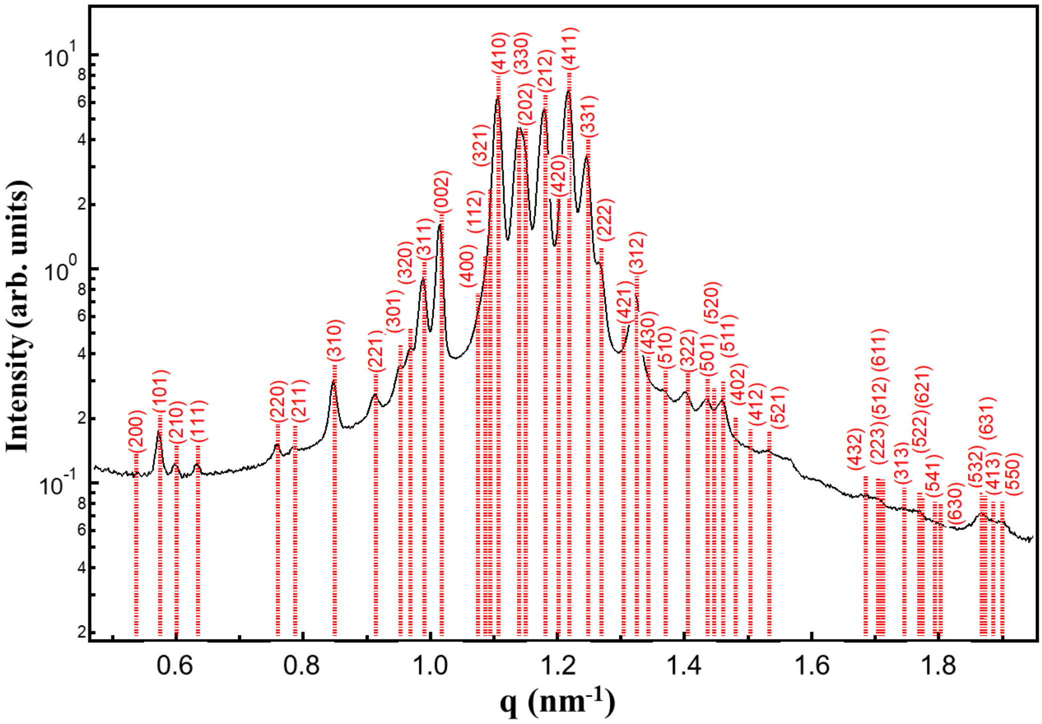

Figure S1. SAXS profile of the G1/CIS 90/10 blend collected at 170 °C, indexed to the characteristic reflections of the Frank–Kasper σ phase (space group $P4_2/mnm$) with lattice parameters a = 23.42 nm and c = 12.37 nm.

3. Temperature-dependent SAXS profiles of G1/CIS 70/30 blends collected during heating and subsequent cooling process from the as-prepared state

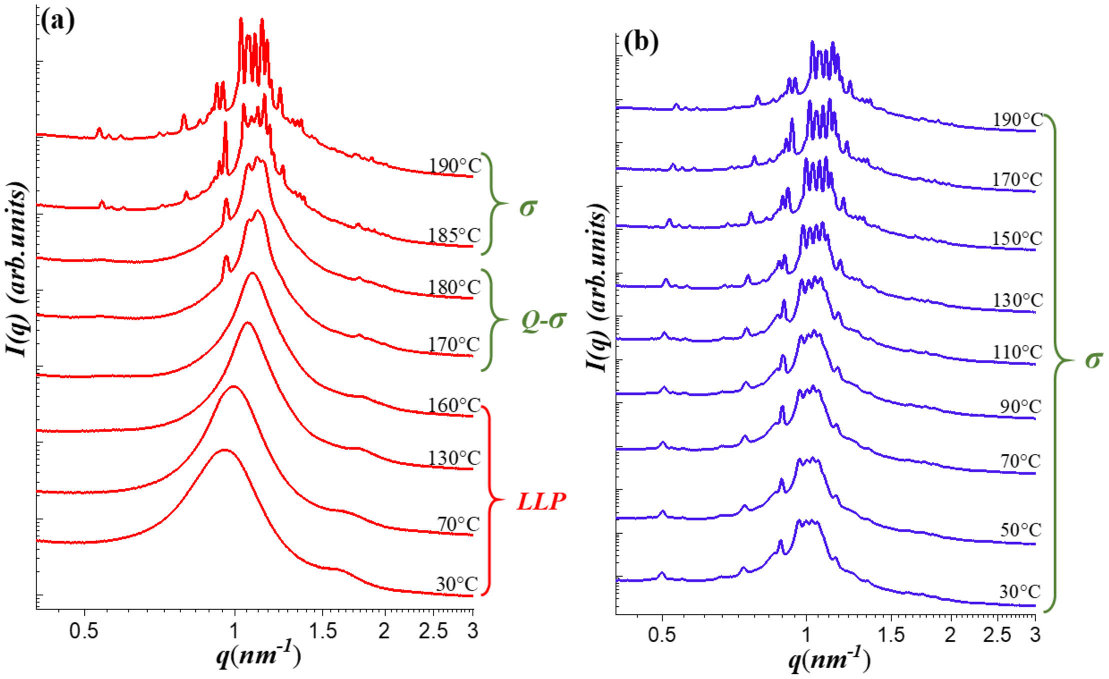

Figure S2. Temperature-dependent SAXS profiles of G1/CIS 70/30 blend at selected temperatures during the (a) heating and (b) cooling process from the as-prepared state. The samples were heated in 10 °C intervals at a ramp rate of approximately 5 °C/min. At each designated temperature, the sample was annealed for five minutes before SAXS data acquisition. The structures formed over the specific temperature ranges are indicated in the figure.

4. Visual evidence of thermally-induced caramelization in the neat CIS after thermal annealing

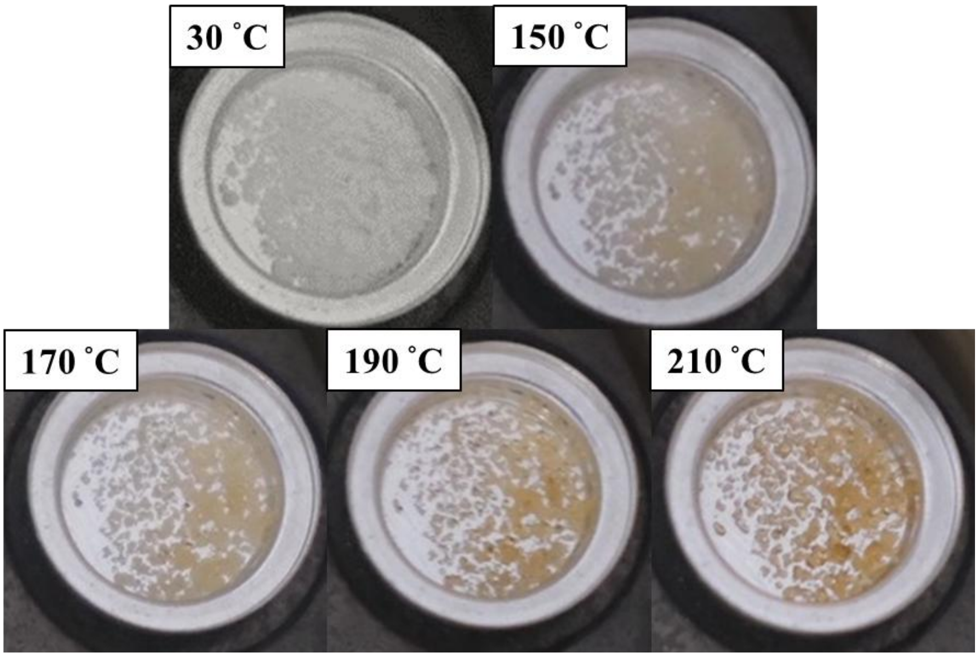

Figure S3. Visual evidence of thermally-induced caramelization in the neat CIS sample after DSC heating, showing the evolution of surface coloration from the pristine white state with increasing annealing temperature. After annealing at 150 °C, a faint yellow tint emerged at the periphery. Annealing at 170 °C produced a more uniform and intensified yellow coloration across the entire surface. Further heating to 190 °C yielded a pronounced amber tone indicative of advanced chromophore formation. Finally, at 210 °C, the sample exhibited a deep brown coloration consistent with extensive caramelization.

5. SAXS profile of G1/CIS 50/50 blend collected at 190 °C, indexed to the characteristic reflections of the Frank–Kasper A15 phase

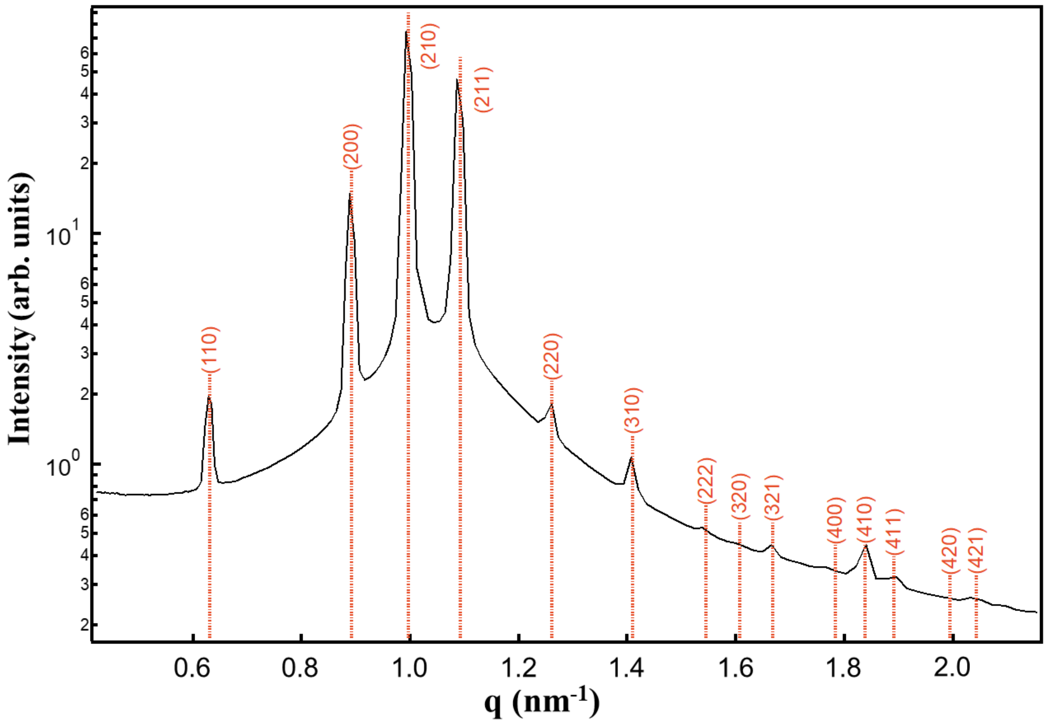

Figure S4. SAXS profile of the G1/CIS 50/50 blend collected at 190 °C, indexed to the characteristic reflections of the Frank–Kasper A15 phase (space group $Pm\bar{3}n$) with a cubic unit cell and lattice parameter a = 14.0 nm.

6. SAXS profile of the G1/CIS 80/20 blend collected during the reheating process to 150 °C, indexed to the characteristic reflections of the Laves C14 phase.

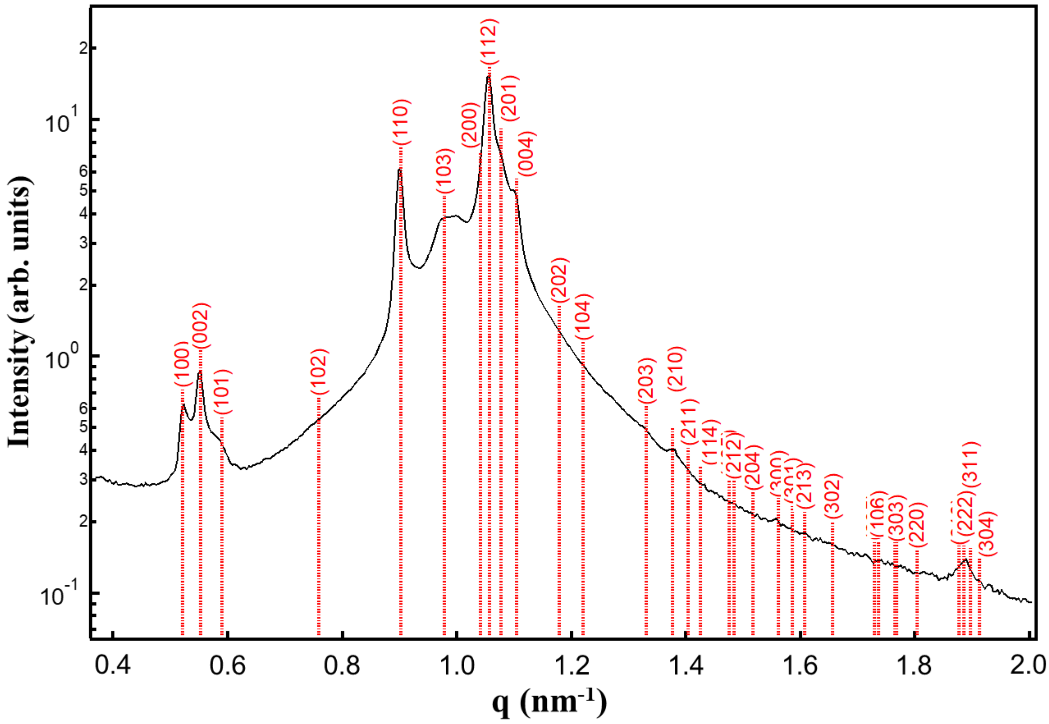

Figure S5. SAXS profile of the G1/CIS 80/20 blend collected during the reheating process at 150 °C, indexed to the characteristic reflections of the Laves C14 phase (space group *P*6$_3$/*mmc*) with lattice parameters *a* = 13.96 nm and *c* = 22.84 nm.

7. SAXS profile of G1/CIS 50/50 blend collected during the reheating processes to 180 °C, indexed to the characteristic reflections of the Laves C15 phase

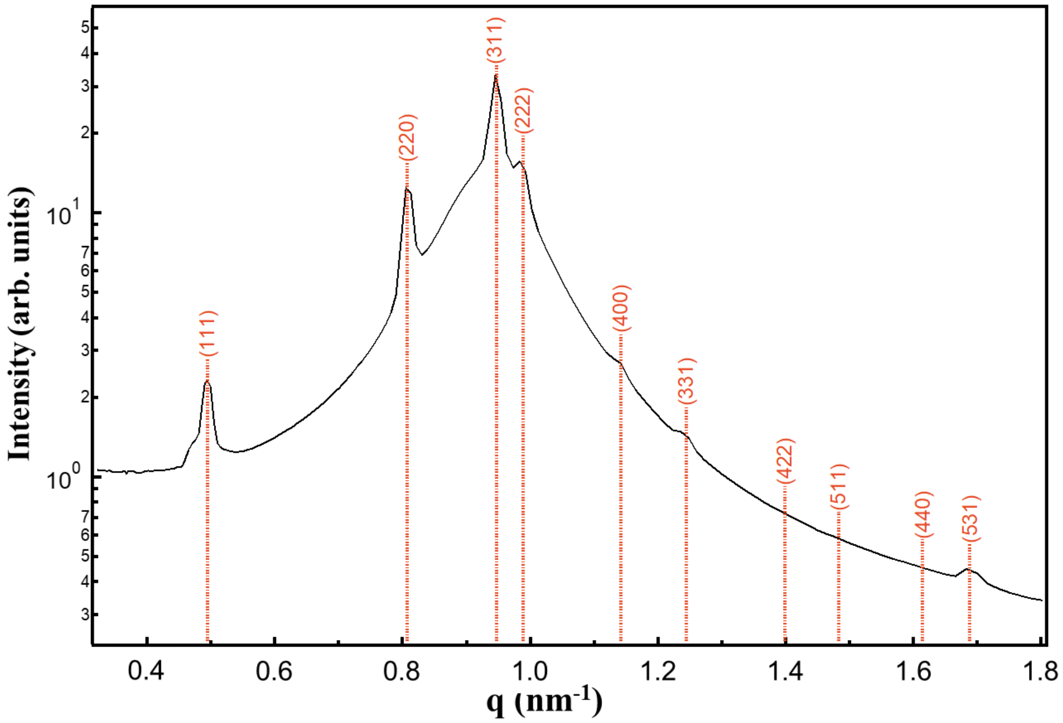

Figure S6. SAXS profile of the G1/CIS 50/50 blend collected during the reheating process at 180 °C, indexed to the characteristic reflections of the Laves C15 phase (space group $Fd\bar{3}m$) with a cubic unit cell and lattice parameter a = 22.1 nm.

8. Temperature-dependent SAXS profiles of G1/CIS 50/50 blends collected during heating and cooling, and reheating process from the as-prepared state under a modified heating protocol.

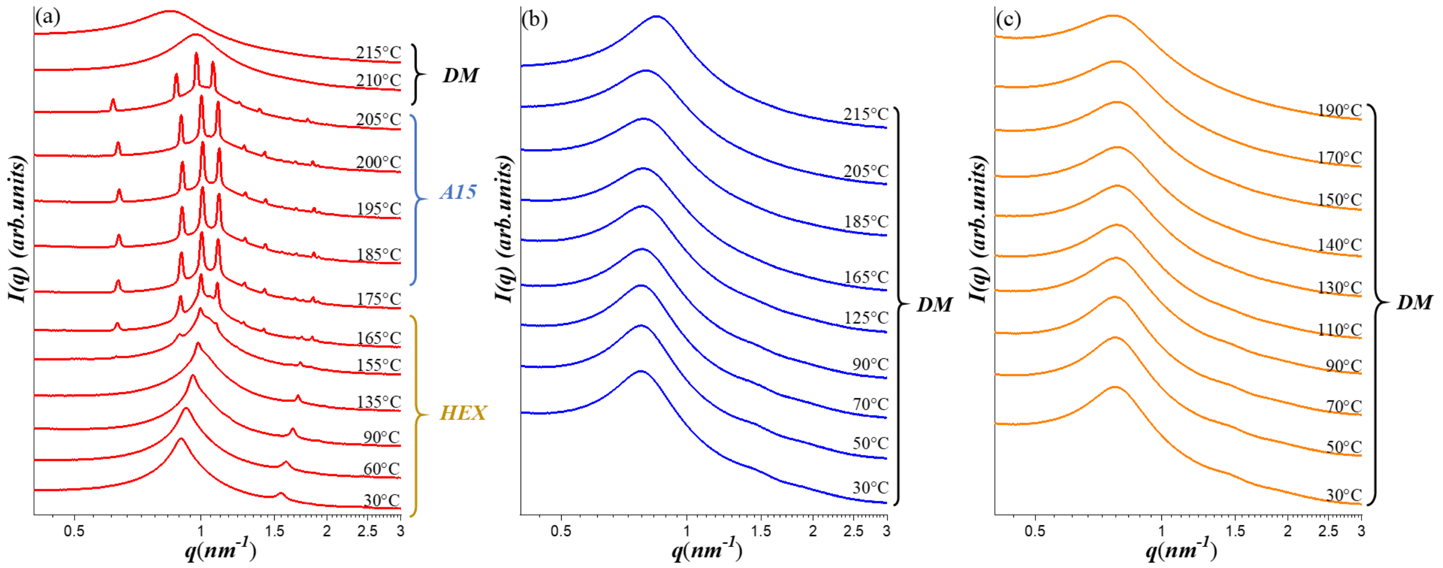

Figure S7. Temperature-dependent SAXS profiles of the G1/CIS 50/50 blend under a modified heating protocol. The sample was gradually heated to 215 °C (5 °C higher than in Figure 3a), followed by cooling and reheating. (a) Upon heating, a distinct shift of scattering peaks toward lower q is observed at 215 °C, indicating micelle swelling and structural disordering due to overheating. (b) Cooling from this overheated state results in disordered micelles with no reformation of Laves-like order. (c) Reheating confirms the irreversible loss of order. These results suggest that Laves phase formation requires carefully controlled thermal history, and excessive heating can destabilize ordered packings.

9. Temperature dependence of the SAXS peak position (q*) and full-width at half-maximum (FWHM) of the LLP phases formed in G1/CIS 80/20 blend.

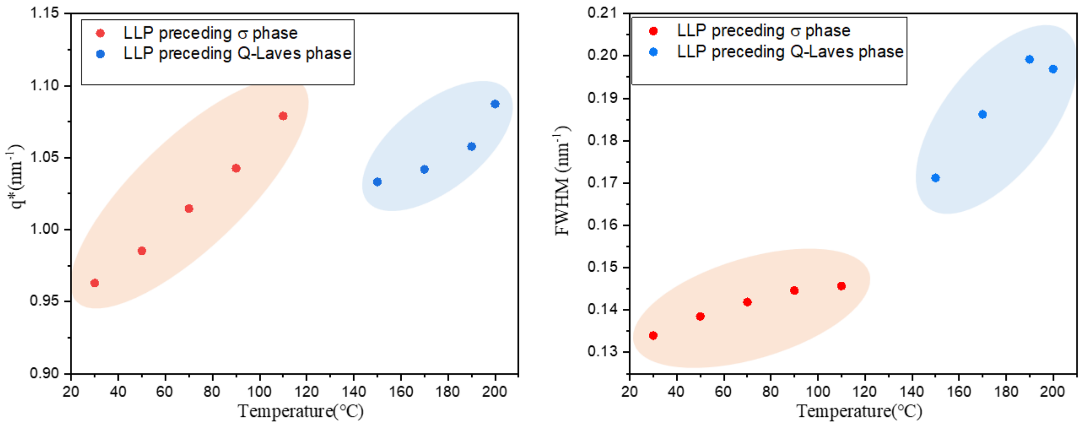

Figure S8 Temperature dependence of the SAXS peak position (q*) and full-width at half-maximum (FWHM) of the LLP phases formed in G1/CIS 80/20 blend. The LLP phase preceding Q-Laves formation exhibits a noticeably smaller q* and a substantially broader FWHM (reflecting larger average micelle size and broader size distribution), whereas the LLP that gives rise to σ displays a smaller q* and a narrower FWHM (indicative of smaller micelle size and narrower size dispersity).
